# Basaloid follicular hamartomas in pediatric Basal Cell Nevus Syndrome: A diagnostic challenge

**DOI:** 10.1111/1346-8138.15892

**Published:** 2021-05-21

**Authors:** Francesca Besagni, Emi Dika, Costantino Ricci, Cosimo Misciali, Giulia Veronesi, Barbara Corti, Carlotta Gurioli, Iria Neri

**Affiliations:** ^1^ Department of Experimental, Diagnostic and Specialty Medicine (DIMES) University of Bologna Bologna Italy; ^2^ Dermatology Unit IRCCS of Azienda Ospedaliero‐Universitaria Policlinico Sant'Orsola Hospital Bologna Italy; ^3^ Pathology Unit Ospedale Maggiore Bologna Italy; ^4^ Pathology Unit S.Orsola Malpighi Hospital Bologna University Bologna Italy

**Keywords:** Basal Cell Nevus Syndrome, basaloid follicular hamartomas, basal cell carcinomas, skin, PTCH

## Abstract

Basal Cell Nevus Syndrome (BCNS) is an autosomal dominant inherited disease caused by *PTCH1* (9q22.3‐q31) germline mutations. Skin manifestations are mainly characterized by hyperkeratosis of the palms and soles, palmoplantar pits and a strong predisposition to develop multiple basal cell carcinomas (BCCs). Recently, it has been hypothesized that basaloid follicular hamartomas (BFH) could be included in BCNS skin features. We present three pediatric cases of GS with BCCs and BFHs. Clinical, dermoscopic and immunohistochemical tools are reported.

**Abbreviations**
AR: Androgen receptor
BCC: basal cell carcinoma
Bcl2: B‐cell lymphoma 2
BCNS: Basal Cell Nevus Syndrome
Ber‐Ep4: Ep‐CAM/cell adhesion molecule epithelial
BFH: basaloid follicular hamartoma
CD34: Cluster of differentiation 34
P: peripheral
P+C: peripheral and central

## 1 INTRODUCTION

Basal Cell Nevus Syndrome (BCNS) is an autosomal dominant inherited disease caused by *PTCH1* germline mutations (OMIM #109400).^1^, ^2^ It is clinically characterized by bone, neurological and ophthalmic anomalies associated with several cutaneous signs such as hyperkeratosis of the palms and soles, palmoplantar pits and a strong predisposition to develop multiple basal cell carcinomas (BCCs).^1^, ^2^ Genotype‐phenotype correlation may vary within carriers of the same *PTCH1* germline mutation even in the same family.^1^, ^2^, ^3^, ^4^, ^5^, ^6^

Recently, it has been hypothesized that basaloid follicular hamartoma (BFH) could be included as a characteristic skin feature of BCNS.^7^ We describe three pediatric cases of BCNS presenting BCCs in association with congenital or early‐onset BFHs.

## 2 METHODS

Patients with BCNS are evaluated in the Rare Disease Outpatient Service, Unit of Dermatology, Department of Experimental, Diagnostic and Specialty Medicine, University of Bologna, Italy.

Clinical, dermoscopic and histological features of BCCs and BFHs were collected for all the selected cases. Routine histological analysis was performed on formalin‐fixed, paraffin‐embedded, 5‐μm thick sections and stained with hematoxylin‐eosin. Immunohistochemistry was performed on 3‐μm thick sections of formalin‐fixed, paraffin‐embedded tissue on the VENTANA BenchMark ULTRA automated slide immunostainer, using the following antibodies: Ber‐EP4 (mouse, monoclonal, clone Ber‐ EP4, dilution ready to use/RTU, catalog no. Ventana 760‐4383, Roche Diagnostics, Rotkreutz, Switzerland), Androgen Receptor‐AR (rabbit, monoclonal, clone SP107, dilution RTU, catalog. No. Ventana 760‐4605, Roche Diagnostics), CD34 (mouse, monoclonal, clone QBEnd/10, dilution RTU, catalog no. Ventana 790‐2927, Roche Diagnostics) and Bcl2 (rabbit, monoclonal, clone SP66, dilution RTU, catalog no. Ventana 790‐4604, Roche Diagnostics). Positive and negative controls were used when appropriate. The slides of the present study were read by two pathologists (C.M., C.R.) with a multiheaded microscope in a nonblinded manner, using a semi‐quantitative immunoscore. Blood samples were drawn from patients after the provision of informed consent.

## 3 RESULTS

### 3.1 Case 1

A 7‐year‐old girl, born to non‐consanguineous parents, was referred to our department for the evaluation of numerous soft brownish papules on her face, trunk and back resembling skin tags. On clinical evaluation we also observed translucent pearly papules, mainly localized on the limbs, and on the dorsum of the feet and toes. Her parents reported that the lesions had appeared after birth. One of the skin tag‐like lesions and one of the translucent papules were surgically removed, with a histological diagnosis of infundibulo‐cystic BCC and BFH, respectively. On careful examination, peculiar facial anomalies such as macrocephaly, broad nasal ridges, heavy and fused eyebrows, mild hypertelorism with down‐slanting eyes and micrognathia were noted. Also, mandibular odontogenic keratocysts had been surgically removed at the age of 5 years. Genetic analysis of the *PTCH1* gene showed c.878_904delTTA de novo mutation in a heterozygote state, confirming our suspicion of sporadic BCNS. Family history was negative for BCNS.

### 3.2 Case 2

A 6‐year‐old Caucasian girl, born to non‐consanguineous parents, was referred to our center with a suspicion of BCNS. She had undergone surgical removal of a medulloblastoma followed by chemotherapy and stem cell transplant at the age of one year and presented a history of BCNS on her father's side. On physical examination numerous erythematous skin tag‐like lesions were observed on her neck and two were removed, with a histological diagnosis of infundibulocystic BCCs. Pearly translucent papules ranging from 1 to 2 mm in diameter on the trunk and the dorsum of foot and toes were also present. On anamnesis, the onset was at the age of six months, with a progressive growth in diameter over the years. Four of these lesions were removed, with a diagnosis of BFHs. Analysis of the *PTCH1* gene confirmed the c.886delT mutation in a heterozygote state of *PTCH1* gene, also present in her father.

### 3.3 Case 3

A 5‐year‐old Caucasian boy, born to non‐consanguineous parents, presented at birth a triventricular hydrocephalus, treated with ventriculoperitoneal repositioning. At the age of 4 years, the patient presented a head circumference in the 97th percentile, mild frontal bossing and synophrys. Radiographic examination revealed odontogenic cysts adjacent to the left mandibular canal. Small hyperpigmented papules, present at birth, were detected on the sole of the right foot. The lesion was removed and the histological diagnosis was BFH. In the next six months, another two similar lesions were removed, with the same histological diagnosis. The child underwent further investigations due to a strong clinical suspicion of sporadic BCNS and the diagnosis was confirmed by the analysis of the *PTCH1* gene, which highlighted a c.2795_2795delT de novo mutation in a heterozygote state. Family history was negative for BCNS.

### 3.4 Clinical and dermoscopic results

**FIGURE 1:**
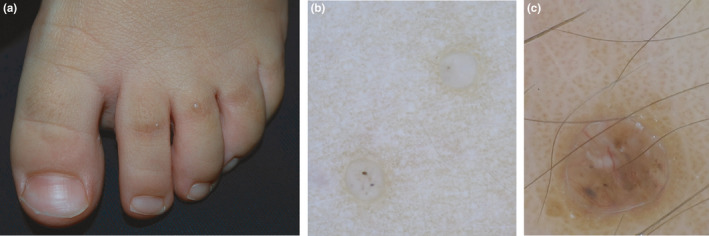
(a) Basaloid follicular hamartomas (BFHs) on the dorsum of the feet presented as skin‐colored translucent papules; (b) dermatoscopic features of BFH presented blue‐gray globules in the absence of vascular structures; (c) dermatoscopic features of basal cell carcinomas showed blue‐gray globules and nests associated with arboriform vessels

In all the presented cases BCCs clinically appeared as brownish or erythematous papules resembling skin tags. BFHs showed pearly translucent skin‐colored or hyperpigmented papules ranging from 1 to 3 mm in diameter (Figure 1a). These lesions were mainly localized on the acral areas, but also on the trunk and limbs. On dermoscopy, the BFHs presented blue‐gray ovoid globules and nests similar to the features of BCC. However, the absence of vascular structures on the background suggested differential diagnosis (Figure 1b–c).

### 3.5 Histological and immunohistochemical results

**FIGURE 2:**
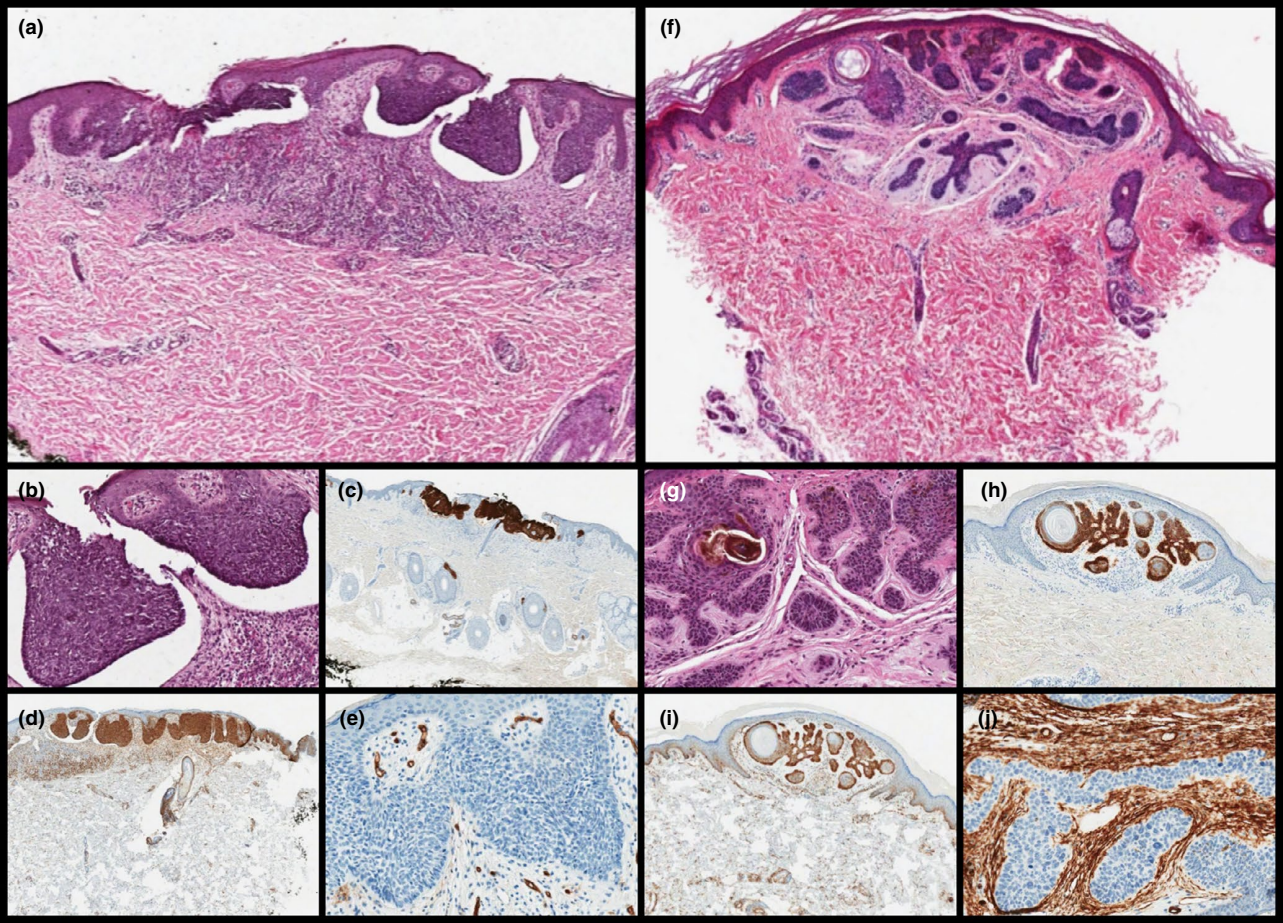
(a–b) Scanning and high magnification shows basal cell carcinoma (BCC) with superficial proliferation of basaloid cells, peripheral palisading and clefts within between the epithelial component and the typical fibrous and mucinous stroma (a, H&E, original magnification ×60; b, original magnification ×200). The lesion was diffusely positivity for (c) Ber‐EP4 (original magnification ×60) and (d) BCL2 (original magnification ×60), (e) with stroma negative for CD34 (original magnification ×200). (f–g) Scanning magnification showed BFH with the so‐called "inverted candlestick" shape, radially emerging from the follicular axis and replacing the affected follicular unit with no destruction of the interfollicular dermis. Note the infundibular cysts with lamellar keratin were inside the basaloid epithelial cords (f, H&E, original magnification ×60; g, H&E, original magnification ×200). The lesion was diffusely positivity for (h) Ber‐EP4 (original magnification ×60) and moderately (with the typical peripheral staining) for (i) BCL2 (original magnification ×60), (j) with stroma negative for CD34 (original magnification ×200)

The histopathological examination of the eight surgically removed BFHs showed two‐three layered epithelial cords adopting the so‐called “inverted candlestick” shape, radially emerging from the follicular axis and replacing the affected follicular unit with no destruction of the interfollicular dermis. Inside the basaloid epithelial cords, frequent infundibular cysts with lamellar keratin were observed. The stroma was scant, with few fibrocytes and fine collagen fibers. On the contrary, the three surgically removed BCCs showed an infiltrative growth pattern with the involvement of the interfollicular dermis and destruction of hair follicles, peripheral palisading and clefts within the epithelial component and the typical fibrous and mucinous stroma. Moreover, numerous mitoses and scattered foci of necrosis were identified in the BCCs. Ber‐Ep4 showed diffuse and strong positivity in both BFH and BCC. Androgen receptor (AR) labeled all the BFHs (7:1+; 1:3+) and BCCs (1:1+; 1:2+, 1:3+) with no clear‐cut differences. The only two antibodies with notable differences between BFH and BCC were Bcl2 and CD34. Bcl2 was diffusely positive (3+) in all BCCs, with no differences between the central and peripheral portion of the lesion, unlike the BFHs (1:1+ and 7:2+, with typical peripheral staining). CD34, evaluated in the stromal component (stromal fibrocytes), stained all the BFHs (2:1+, 2:2+; 4:3+) and was completely negative in all the BCCs. Immunohistochemical data are summarized in Table1 together with the criteria used for the immunoscore. Some histological and immunohistochemical results are shown in Figure 2

**TABLE 1:** Immunohistochemical data of BFHs and BCCs

Patient number	Histological diagnosis	CD34	BCL2	Ber‐EP4	AR
1	BFH	3	2 (P)	3	1
	BCC infundibolo‐cystic	0	3 (P+C)	3	2
2	BCC superficial	0	3 (P+C)	3	3
	BCC superficial	0	3 (P+C)	3	1
	BFH	3	2 (P)	3	1
	BFH	1	2 (P)	3	1
	BFH	3	2 (P)	3	3
	BFH	1	2 (P)	3	1
3	BFH	2	2 (P)	3	1
	BFH	2	2 (P)	3	1
	BFH	3	1 (P)	3	1

Note

Score 0 (no positive cells); 1 (small fraction of positive cells); 2 (moderate fraction of positive cells); 3 (big fraction of positive cells).

Abbreviations: AR, Androgen receptor; BCC, basal cell carcinoma; Bcl2, B‐cell lymphoma 2; Ber‐Ep4, Ep‐CAM/cell adhesion molecule epithelial; BFH, basaloid follicular hamartoma; CD34, Cluster of differentiation 34; P, peripheral; P+C, peripheral and central.

## 4 DISCUSSION

Basal Cell Nevus Syndrome is an autosomal dominant disorder caused by *PTCH1* germline mutations, which mapped to chromosome 9q22.3‐31.^1^ *PTCH1* acts as an oncosuppressor gene, predisposing affected patients to the development of multiple BCCs that represent the cutaneous hallmark in this disease. BCCs occur more frequently after puberty, although in some cases may appear earlier in childhood. The peculiar morphology of BCC in children has been described as flesh‐colored and pigmented polypoid lesions, clinically resembling skin tags.^3^, ^4^, ^5^ Other authors have described the early onset of nodular BCCs with acral distribution.^4^, ^5^, ^6^ A correct diagnosis of BCCs in pediatric age and the differential diagnosis with numerous benign adnexal tumors and hamartomas is fundamental.^8^ Recently, it has been hypothesized that basaloid follicular hamartoma (BFH) could be included as a characteristic skin feature of BCNS.^7^ BFH is a rare follicular malformation usually appearing as a cyst, plaque or small skin‐colored papule, and often clinically misdiagnosed.^8^ Several studies have linked the PTCH1 mutation to the formation of BFH, as well as BCC in the context of BCNS.^8^ This underlines the clinical impact of BHF, which could influence the early diagnosis of BCNS, also in the absence of the classic skin phenotype. It is plausible to hypothesize that BCC and BFH represent a different spectrum of the same genetic disease. The development of basal cell carcinoma occurring within BFH lesions has been reported, however further studies are yet to be performed.

In our series, the early onset of several BFHs is reported in BCNS. Clinically, small papules involved the dorsum, the lateral side of the hands and feet, the trunk, the face and the neck. A combination of clinical, dermatoscopic, histopathological and immunohistological data were analysed to perform the correct diagnosis.

Dermoscopy could improve the evaluation of differential diagnosis between BCC and BFH, especially in pediatric cases when BCNS is suspected. We evaluated the diagnostic criteria for BCC proposed by Menzies et al.^9^ and found that achrocordon‐like BCCs showed small blue‐gray ovoid nests and fine vessels contouring the peduncle, in the absence of larger diameter vessels surmounting the lesions as described by Rodriguez et al.^5^ In our experience, dermoscopy of the BFHs revealed only the presence blue‐gray globules, in the absence of associated vascular structures pathognomonically described also in small diameter BCCs.^10^

Regarding laboratory studies, recent immunohistochemical studies have suggested the use of single antibodies such as ki‐ 67, Ber‐EP4, CD34,CK 20, Bcl2 and AR to differentiate BCC from BFH. Hence, the results are not standardized.^11^, ^12^, ^13^, ^14^, ^15^, ^16^ We believe that the use of an immunohistochemical panel including Ber‐EP4, CD34 and Bcl2 and AR, could be useful in the differential diagnosis of adnexal neoplasms, BCC and mimickers, especially in small biopsies where the evaluation of all histological criteria could be limited.^17^ In particular, our study proved that the two antibodies with notable differences between BFH and BCC were Bcl2 and CD34. Bcl2 was diffusely positive in all BCCs with no differences between the central and peripheral zone; while CD34 evaluated in the stromal component stained all the BFHs and was completely negative in the BCCs.

In conclusion: (i) This study supports the potential link between BFH and BCNS; (ii) dermoscopic features could be useful in the differential diagnostic of BFH /BCC (iii) an immunohistochemical panel comprising CD34 and Bcl2 differentiated BFH from BCC.

In our experience, in line with the literature data, BFHs in BCNS may appear congenitally or early in life. Given their observation only in pediatric BCNS patients but not in adults, a possible involution over the years could be hypothesized and the need for aggressive treatment in pediatric patients is therefore questionable. A validation of these results on larger case series should be encouraged, as a wait‐and‐see approach might be suggested in these cases.

## CONFLICT OF INTEREST

None declared.
